# A sensitivity study of rising compound coastal inundation over large flood plains in a changing climate

**DOI:** 10.1038/s41598-022-07010-z

**Published:** 2022-03-01

**Authors:** Y. Peter Sheng, Vladimir A. Paramygin, Kun Yang, Adail A. Rivera-Nieves

**Affiliations:** Coastal and Oceanographic Engineering Program, University of Florida, Gainesville, FL 32611-6580 USA

**Keywords:** Climate sciences, Hydrology, Natural hazards, Ocean sciences, Engineering, Mathematics and computing

## Abstract

Coastal flood hazards and damage to coastal communities are increasing steeply and nonlinearly due to the compound impact of intensifying tropical cyclones (TCs) and accelerating sea-level rise (SLR). We expand the probabilistic coastal flood hazard analysis framework to facilitate coastal adaptation by simulating the compound impact of predicted intensifying TCs and rising sea levels in the twenty-first century. We compared the characteristics of landfalling TCs in Florida (FL) and southwest Florida (SWFL) for the late twentieth and twenty-first centuries predicted by several climate models and downscaling models. TCs predicted by four climate models, one without downscaling and three with downscaling, were used by a coupled surge-wave model to predict the future flood hazard due to compound effects of TCs and SLR over a large SWFL coastal flood plain. By 2100, the coastal inundation metrics of the 1% annual chance coastal flood could become almost 3–7 folds of their current values, depending on the climate and downscaling models, Representative Concentration Pathway scenarios, Atlantic Multi-decadal Oscillation phases, TCs, SLR, precipitation, and how TCs and SLR are incorporated. By 2100, the current 1% (100-year) inundation event could become a 3-year event, and the 0.2% (500-year) inundation event could become a 5-year event.

## Introduction

Coastal flood vulnerability has increased worldwide due to gradually accelerating sea-level rise (SLR)^1–4^, intensifying and wetter tropical cyclones (TCs)^5,6^ driven by climate change and increasing coastal development. Coastal communities have incurred dramatically increasing TC-induced flood damages^7^, which are highly dependent on the local conditions of the flood, wave, infrastructure, SLR, landscape, and their interactions^8^. While 40% of the world population lives in the coastal region within 100 km of the coastline, 10% of the population lives within the low elevation coastal zone (LECZ) less than 10 m above the sea-level^9^. In Florida, 76.5% of the population lives in the coastal region^10^. LECZ constitutes 98.37% of Collier County in southwest Florida. Of the 258 U.S. weather disasters during1980-2020, tropical cyclones caused the most damage: $945.9 billion total, with an average cost of almost $21.5 billion per event^11^. The expected annual loss of residential properties due to hurricanes is $34B, out of which $20B is due to flood^12^. Florida suffered catastrophic losses due to many major hurricanes, including Andrew^13^, Charley^14–16^, Ivan^17^, Wilma^16,18^, Irma^19^, and Michael^19^ in the past three decades. The flood-induced property loss is expected to increase dramatically in the following decades. Therefore, regional and local scale adaptation plans need to be developed quickly to minimize future flood damage based on the best understanding of how a changing climate will impact the TCs and SLR and coastal flooding in the twenty-first century.

To facilitate coastal adaptation planning, the status quo probabilistic coastal flood analysis framework^9^, which considers the 1% coastal flooding due to an ensemble of synthetic TCs based on historical hurricane data^20^, needs to be expanded to incorporate the compound effects of TCs and SLR in a changing climate. Studies on future 1% coastal flooding over a large coastal flood plain, which are needed for adaptation planning by coastal communities, are scarce^21,22^. Studies of the impact of TCs and SLR on future 1% storm surge at coastal stations or impact of SLR on a single TC^23,24^ do not provide sufficient spatial detail of coastal flooding for adaptation planning by coastal communities.

While it is known that SLR is directly increasing nuisance tide flooding and groundwater flooding, the rising sea-level and warmer ocean and atmosphere also appear to be causing more intense and wetter tropical cyclones. Projection of future coastal flood hazards requires the prediction of future tropical cyclones by climate models, which generally have a relatively coarse resolution (~ 25–50 km), and downscaling models. A group of leading climate and hurricane scientists reached a consensus estimate that, by 2100, globally averaged hurricane wind intensity will increase by 2–11%, and the globally-averaged frequency of hurricanes will decrease by 6–34%^4^. Globally, it is about 25% more likely now that a TC will be at major TC intensity (Category 3, 4, 5) than four decades ago^25^. In the Atlantic, the proportion has more than doubled. Almost all mortality and damage are caused by major TCs^26^. A recent study^6^ expressed high confidence that the global average TC intensity will increase and the global proportion of very intense (category 4–5) TCs will increase. The median projected change is about + 13%. These intense TCs are responsible for the greatest damage and mortality rates.

While our understanding of the role of climate change in affecting TCs over global and basin scales has progressed in the past two decades, there is limited understanding of the future TCs and their impacts on coastal flood hazards due to inadequate data and modeling studies. Adding to the uncertainties of the coastal surge and wave models and data for predicting coastal flooding^14,27^, considerable uncertainties are associated with predicting future TCs by climate and downscaling models^28–30^, particularly on the regional and local scales. Uncertainties arise from the different parameterization of the important physical processes and resolution of the climate and downscaling models. A comprehensive uncertainty analysis surrounding the prediction of future TCs requires an excessive amount of data that are unavailable and beyond the scope of this study. Instead, this study focus on the sensitivity of future probabilistic flood hazard over a large coastal floodplain in SW FL to several important factors: SLR, future TCs predicted by various climate and downscaling models, Representative Concentration Pathway (RCP) scenarios, Atlantic Multidecadal Oscillation (AMO) phases, precipitation, and how SLR and TCs are combined in coastal model simulations. We hope to develop a new paradigm for assessing future coastal flood hazards in coastal regions impacted by TCs and SLR globally.

**Figure 1:**
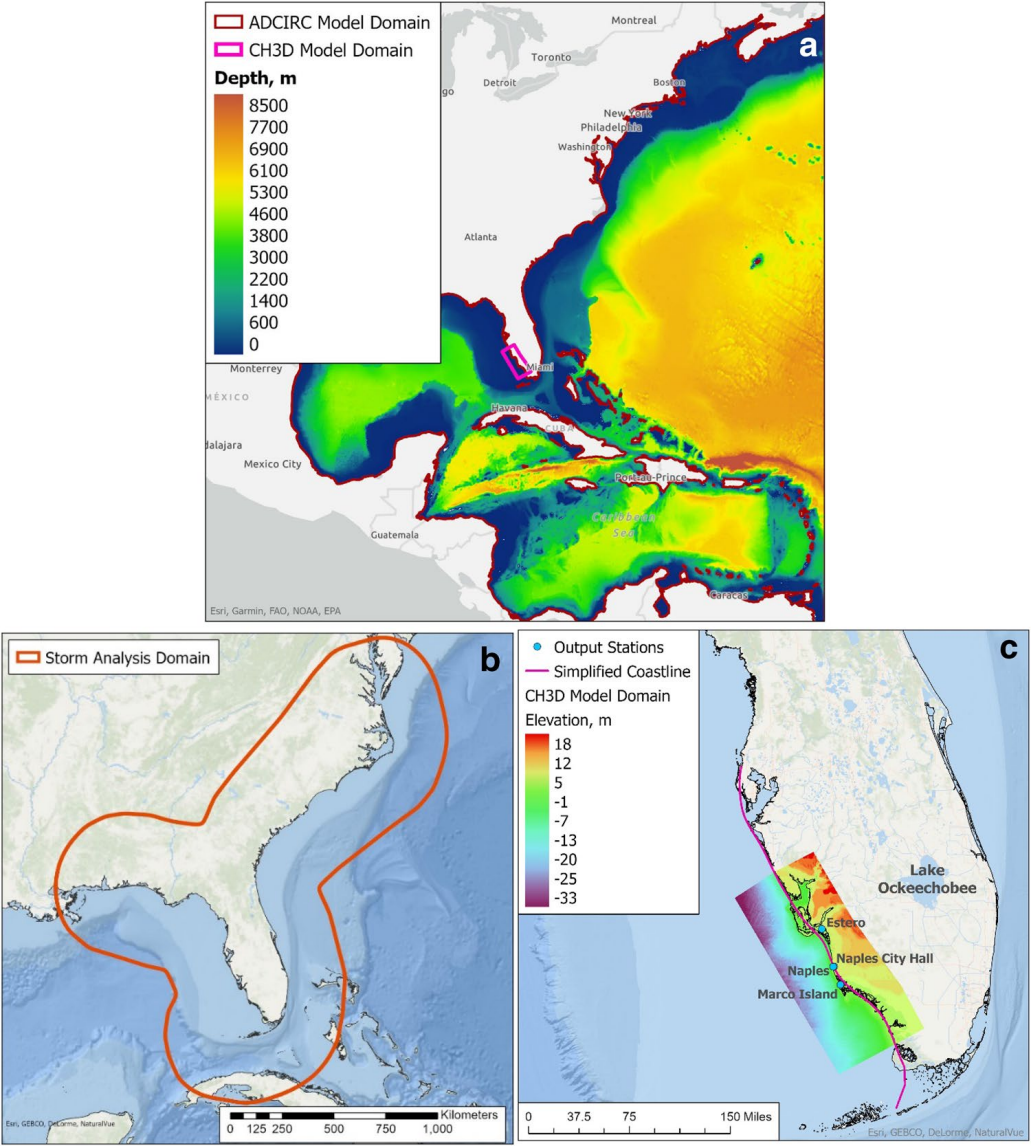
(**a**) SWFL model domain inside the large ADCIRC or CH3D domain; (**b**) Southeastern US domain used by Liu et al. (2014) for TC downscaling analysis with 12-km WRF; (**c**) SWFL model domain with idealized coastline for JPM and CH3D-SWAN model output stations. Elevations are shown with respect to NAVD88. Gentle bathymetric and topographic slope (~ 1/1000) is much lower than that in Southeast Florida. The simplified coastline is 425 km long, constituting 20% of Florida’s coastline and exceeding the combined coastlines of NJ and NY.

We examine the feasibility and sensitivity of using TC predictions by climate models with relatively coarse model resolution (> 50 km for most global climate models and 25 km for high-resolution global climate models) and downscaling models with very different techniques for coastal flood analysis over 425 km long coastline in SWFL which exceeds the combined coastlines of New Jersey and New York (Fig. 1). With the 83rd percentile SLR at Naples reaching 2.54 m by 2100^1,2^, high TC landfall rate, intensifying TCs^5,6^, a flat bathymetry and topography, and a rapidly growing population, SWFL ranks among the most vulnerable US coasts for future flooding. In addition, the very gentle bottom slope of ~ 1/1000 for 100 km offshore makes SWFL more prone to flood impact during storms versus southeast Florida with a much steeper bottom slope. Southwest Florida (SWFL, including Charlotte, Lee, and Collier Counties), home to more than 1.3 million people, is the second most hurricane-prone area in Florida with 49 historical hurricanes and two of the top 10 costliest hurricanes in the US ($30B for Irma in 2017 and $11B for Wilma in 2005).

In the following methods section, we describe how future TCs and SLR scenarios are selected, the coastal surge and wave models used for simulating coastal flooding during individual TC&SLR combinations, and the statistical method used for determining the 1% coastal flood elevation over the large coastal flood plain. We then present the 1% coastal flood results in 2020, 2030, 2060, and 2100 in terms of the evolution of four major coastal inundation metrics. Accomplishments and future studies are then discussed.

## Methods

### Selection of tropical cyclones for the twenty-first century

We consider TC datasets predicted by three global climate models (GCMs) (CESM^30^, HiRAM-SITHR^31^, and CAM5.1^32^) without downscaling, and six global climate models (CAM5.3^33^, HiRAM^34^, HADGEM^26^, GFDL5[GFDL’s GCM for CMIP5, i.e., Coupled Model Intercomparison Project Phase 5]^35^, and GFDL6 [GFDL’s GCM for CMIP6, i.e., Coupled Model Intercomparison Project Phase 6]^36^, and FSUGSM^37^ with four very different downscaling models (NASHM^38^ for CAM5.3, GFDL Hurricane Model^39^ for HiRAM, WRF^40^ for FSUSGM, and KE [Kerry Emanuel’s model]^41^ for the others), and two historical TC datasets (HURDAT^20^ and NCEP2^42^ reanalysis). The four downscaling models are very different: NASHM is a stochastic model with 1000 repeats of the global model simulation period, GFDL’s hurricane model is a regional climate model (RCM), a regional scale WRF with a 12-km resolution was used to downscale the results of the global FSUGSM model, while KE is a statistical-deterministic model forced by environmental conditions independent of the TCs predicted by the global climate models as the other two downscaling models do.

Except for the historical datasets, all datasets are associated with different times (e.g., late twentieth century and early and/or late twenty-first century), AMO phases (positive and negative), and RCP scenarios (4.5 and 8.5). Here we examine the sensitivity of changes in landfalling TC characteristics in SWFL and FL in the twenty-first century to climate and downscaling models, AMO phases, and RCP scenarios. We focus on the predicted changes of three dominant landfalling TC characteristics: central pressure deficit (∆Cp), radius of maximum wind (R_max_), and TC translation velocity (V_f_), before selecting TC datasets for coastal flooding analysis. The naming convention of the TC datasets is described in the caption of Fig. SI-1.

### Coupled hydrodynamic and wave models

Curvilinear-grid hydrodynamics in 3D (CH3D) is a hydrodynamic model originally developed by Sheng^43,44^, which can simulate 2-D and 3-D barotropic and baroclinic circulation driven by tide, wind, and density gradients. CH3D is more efficient than many high-fidelity hydrodynamic models (e.g., ADCIRC^45^, POM^46^, and FVCOM^47^) using a semi-implicit numerical algorithm for similar grid resolution^14,48^. CH3D is dynamically coupled to SWAN^49^ to simulate surge, wave, and flooding in coastal regions. In addition, basin-scale ADCIRC was used to provide offshore open boundary conditions. This integrated storm surge modeling system^18,46^ can simulate flooding and drying with non-linear terms and has been used successfully to simulate storm surges along the US Atlantic and Gulf coasts during Charley, Wilma, and Irma, which significantly impacted SWFL^13–19^. Details of the CH3D model and the SWAN model can be found in numerous papers in the references, particularly Sheng et al.^48^ and Booij et al.^49^; they will not be repeated here.

The coupled surge-wave model CH3D-SWAN, driven by a synthetic parametric wind model^50^, was used with the Joint-Probability-Method with Optimal Sampling (JPM-OS)^21,51–53^. First, the wind and atmospheric pressure fields were calculated based on the location of the storm, pressure at the center, radius to maximum winds, and translational velocity of the storm. Next, an ensemble of straight-line storm tracks, with all parameters kept constant until landfall and then allowed to decrease, was generated^53^. Finally, storms were simulated by a 2D vertically averaged version of CH3D-SWAN, which accurately (with 10% of relative Root Mean Square Error) simulated the observed High Water Marks (HWMs) in the region during Hurricane Irma^19^.

The SWFL domain (Fig. 1b) contains 386,140 curvilinear grid cells, minimum resolution of 20 m and average resolution of 200 m, with spatially varying Manning’s coefficients^53^, defined based on the USGS NLCD2011 dataset with a value of 0.02 in the open water. GEOphysical DAta System (GEODAS) and USGS National Elevation Dataset were used as the source of bathymetric (90 m horizontal resolution) and topographic (5–10 m horizontal resolution) data referenced to NAVD88. The flood (or inundation) elevation is the difference between the water level and the local topography. Along the open coastal boundary, the water level is the superposition of the surge simulated by ADCIRC plus the SLR value. The wetting-and-drying condition is applied with zero pressure gradient in the perpendicular direction along the land boundary without prescribing any SLR value.

To reduce the computational effort, this study used the vertically-averaged version of CH3D-SWAN in which land-use features (buildings, mangroves, marshes, etc.) and bathymetric features represented by a spatially-varying Manning’s coefficient for bottom friction.

To examine the effect of precipitation, we used the precipitation data from NOAA’s probabilistic precipitation estimates for the region for simplicity. The 1% precipitation at Naples was 16 inches over three days for the current climate. For 2080–2100, the 0.2% rainfall data of 22 inches over three days were utilized to reflect the increase in precipitation in future climates. Precipitation is applied uniformly over the model domain during each model simulation of a synthetic TC.

### Statistical method (JPM, JPM-OS, and MCLC)

The JPM statistical method allows the generation of a set of all possible TCs according to the joint probabilities of the TCs predicted by the climate and downscaling models for a coastal region during a specific time period (e.g., 20 years). The JPM uses the probabilistic descriptions of landfall parameters and storm rate to determine a set of synthetic storms called test storms. A single storm with a set of the five landfall parameters is called a “node ($x$)” in five-dimensional parameter space:

1 $$\begin{array}{c}x = \left[{p}_{c}\, {R}_{m}\, \theta {V}_{f}\, {L}_{0}\right]\end{array}$$

The storms are simulated with a numerical model to generate the peak water level height $\eta (x)$ for the model domain. The annual rate of occurrence of the water level greater than or equal to a specific value $\eta$ for a cell inside the model domain is calculated by the JPM integral:

2 $$\begin{array}{c}P\left[{\eta }_{max} > \eta \right]= \lambda \int \dots \underset{x}{\overset{ }{\int }}{f}_{X}\left(x\right)P\left[\eta \left(x\right)> \eta \right]dx\end{array}$$

The integral depends on the mean annual rate $\lambda$ of all storms for the domain, the joint probability density function ${f}_{X}(x)$, and the conditional probability that a particular set of storm characteristics ${x}_{i}$ will generate a water level height greater than $\eta$, is $P[\eta ({x}_{i}) > \eta ]$. The integral is evaluated for all test storms, and the annual probability, which has the unit of storms per unit time, is calculated. The annual probability for the storms to produce water level exceeding a value $\eta$ can be approximately calculated by

3 $$\begin{array}{c}P\left[{\eta }_{max} > \eta \right]\approx \sum_{i=1}^{n} {\lambda }_{i} P[\eta ({x}_{i}) > \eta ]\end{array}$$

where $n$ is the number of the test storms, and $P$ is the probability of the storm with the landfall characteristics ${x}_{i}$. Typically, the 1% annual chance flood (also known as the 100-year flood) elevation at any given location is exceeded by the flood elevation during hundreds of low-frequency storms with a cumulative frequency of 1%.

In JPM-OS, a few hundred “optimal” (representative) storms are selected from the JPM test storms by analyzing the discrete probability distribution functions (pdfs) of the five storm characteristics. A surge model then simulates the optimal storms to determine the corresponding peak water levels, which are then interpolated to obtain the peak water levels for all test storms, and probabilistic flood maps for different return periods are calculated.

Various versions of the JPM-OS^51–53^, use different optimization schemes. The merits and shortcomings of JPM-OS methods were compared by Yang et al.^53^, who developed an objective and efficient JPM-OS based on kriging to significantly improve the previous accuracy, efficiency, and objectiveness of JPM-OS methods. They showed that the 1% flood maps produced with 300+ optimal storms were nearly identical to that produced from the 20,625 possible storms described by the discrete pdfs of five major storm characteristics. Furthermore, as explained in Yang et al.^53^, the effect of tide on the 1% flood map is relatively small, partially due to relatively small tides in the region. The JPM-OS can be found in Yang et al.^53^; hence it will not be repeated here. In this study, for each of the 20-year periods and based on the predicted TCs, 190 optimal storms were developed for simulation of probabilistic flood with a specific SLR value prescribed at the open boundary of the coastal model.

While most of the coastal inundation results were obtained using the JMP-OS, we also applied the Rapid Forecast and Mapping System (RFMS)^19^ along with the Monte Carlo life-cycle (MCLC)^54^, which was repeated 10,000 times, to calculate the coastal inundation metrics due to a TC that induced a 1% annual chance Naples water level, and another TC that produced a 1% TIV (50 percentile) in the model domain. The coastal inundation metrics from these two simulations are compared with those obtained with CH3D-SWAN and JPM-OS.

### Coastal inundation metrics

The 1% annual chance inundation metrics, including Total Inundation Volume (*TIV*), Total Inundation Area (*TIA*), Maximum Inundation Height (*MIH*), and Averaged Inundation Height (*AIH*), for the scenarios listed in Table 1 are shown in Fig. 3. The $TIA$ and $TIV$ are defined as^13^:

4 $$TIA=\int {\int }_{Landward\, Area}dxdy$$

5 $$TIV=\int {\int }_{Landward\, Area}[{H}_{max}\left(x,y\right)-{H}_{0}\left(x,y\right)]dxdy$$

where ${H}_{max}(x,y)$ and ${H}_{0}(x,y)$ are the maximum water level and the land elevation at land cells $(x,y)$, respectively for each scenario. *MIH* = Maximum Inundation Height while *AIH* is the average value of *MIH* over the entire domain. These statistics have been used as objective metrics to quantify TC-induced flood hazard over a coastal flood plain by a single TC and the 1% flood due to a TC ensemble^19,53,55^.

**Table 1:** Scenarios for coastal inundation simulations.

	Simulation scenarios	Time period	RCP	AMO phase	TCs	SLR (m)	Percentile of SLR value (Sweet et al.^2^)
A	FSUGSM-WRF-20	1982–2009 (CC)	n/a	n/a	6	0.0	–
B	CAM5.1–20	1995–2005 (CC)	n/a	n/a	12	0.0	–
C	HiRAM-GFDL-20	1981–2000 (CC)	n/a	n/a	7	0.0	–
D	GFDL6-ESM4-KE-20	1850–2014 (CC)	n/a	n/a	> 180	0.0	–
E	FSUGSM-WRF-E21-N	2020–2039 (2040)	4.5	Negative	4	0.0	0th
F	FSUGSM-WRF-E21-N	2020–2039 (2040)	4.5	Negative	4	0.3	99th
G	FSUGSM-WRF-E21-P	2020–2039 (2040)	4.5	Positive	5	0.0	0th
H	FSUGSM-WRF-E21-P	2020–2039 (2040)	4.5	Positive	5	0.3	99th
I	FSUGSM-WRF-L21-N	2080–2099 (2100)	4.5	Negative	9	1.0	83rd
J	FSUGSM-WRF-L21-P	2080–2099 (2100)	4.5	Positive	9	1.0	83rd
K	FSUGSM-WRF-L21-8.5	2080–2099 (2100)	8.5	Positive	9	0.0	0th
L	FSUGSM-WRF-L21-8.5	2080–2099 (2100)	8.5	Positive	9	1.0	83rd
M	FSUGSM-WRF-L21-8.5	2080–2099 (2100)	8.5	Positive	9	2.0	99.7th
N	CAM5.1-L21-8.5	2079–2099 (2100)	8.5	n/a	12	1.0	83rd
O	HiRAM-GFDL-L21-4.5	2080–2099 (2100)	4.5	n/a	7	1.0	83rd
P	GFDL6-ESM4-KE-L21	2015–2100 (2100)	4.5	n/a	> 180	1.0	83rd

### 1% and 0.2% water level at Naples tide station for model verification

To verify the 1% flood calculation, we compared the model simulated probabilistic water level at Naples tide gauge based on the TCs generated by FSUGSM-WRF for the current climate vs. those generated by the peak-over-threshold (POT) method^56^ from the historical hourly water level data from 1965 to 2019. The peak-over-threshold method first detrends the hourly observed water level data, computes the daily maxima of water level, and selects a threshold corresponding to the 99th percentile (after trial and error) of observed water levels. Then the threshold exceedances are fitted to a generalized Pareto distribution (GPD), and the return periods are determined from the distribution. The 1% and 0.2% water levels at Naples are (1.67 m, 1.53 m) from the model and data, respectively, while the 0.2% water levels are (2.47 m, 2.24 m), respectively. The good agreement between the 1% and 0.2% water levels calculated by the model and data indicates the robustness of the model simulation and statistical method.

## Results

### Characteristics of future and historical TCs

Figure SI-1a and SI-1b show ∆Cp values for landfalling TCs in FL and SWFL predicted by the above-mentioned climate and downscaling models as well as from historical data. ∆Cp values predicted by various climate and downscaling models for FL (Fig. SI-1a) and SWFL (Fig. SI-1b) generally increase from the late twentieth century to the late twenty-first century, except those obtained by CESM and HiRAM-GFDL. FSUGSM-WRF and GFDL6-ESM4-KE results show a significant increase in ∆Cp over the twenty-first century. According to the FSUGSM-WRF, CAM5.1, and GFDL-KE results, in the late twenty-first century, ∆Cp and R_max_ for RCP8.5 are lower than those during RCP4.5, perhaps due to stabilization of the upper atmosphere due to excessive warming. ∆Cp and R_max_ also show slight increases during the positive AMO phase. R_max_ values for landfalling TCs in FL (Fig. SI-1c) and SWFL (Fig. SI-1d) show wider variation over time, with an increase predicted by GFDL5-KE and GFDL6-KE, decrease predicted by FSUGSM-WRF (but increase for 75th percentile and higher R_max_), and little change by other models. R_max_ is unavailable from CESM and HiRAM. As shown in Fig. SI-1e and SI-f, the translation velocity (V_f_) changed relatively little over time, in which some models showed a slight decrease while others showed a slight increase. The overall patterns for FL and SW-FL are generally consistent, with a few exceptions.

It should be noted that FSUGSM-WRF-E21 and FSUGSM-WRF-L21 include the results for positive and negative AMO phases. Each scenario covers a 20-year period, so combining the TCs from positive and negative AMOs would double the number of TCs for each period. Compared with the ∆Cp and Rmax for historical TCs, GFDL-KE and FSUGSM-WRF predicted lower ∆Cp and larger Rmax for the twentieth century.

**Figure 2:**
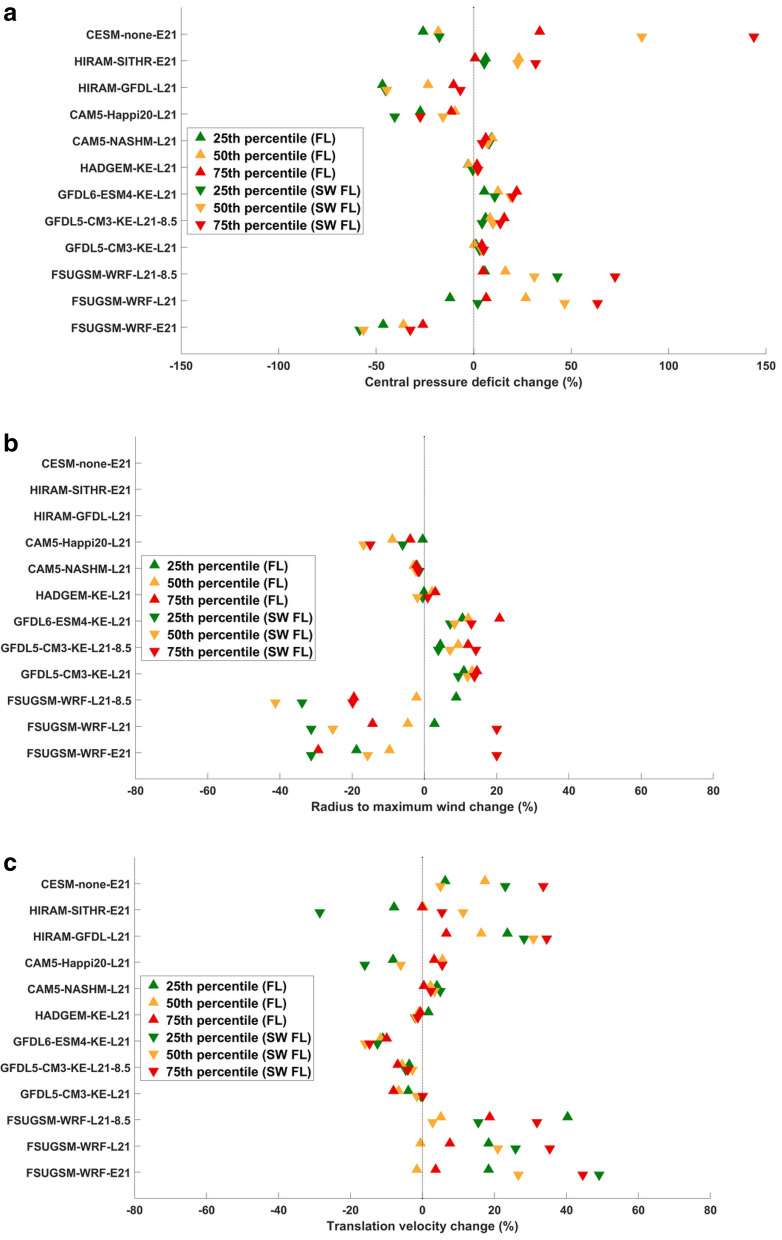
(**a**) Percent change in various percentiles (25th, 50th, and 75th) of ∆Cp over its respective value in the twentieth century in FL (triangles) and SWFL (inverted triangles). (**b**) Percent change in various percentiles (25th, 50th, and 75th) of R_max_ over its respective value in the twentieth century in FL (triangles) and SWFL (inverted triangles). (**c**) Percent change in various percentiles (25th, 50th, and 75th) of V_f_ over its respective value in the twentieth century in FL (triangles) and SWFL (inverted triangles).

To summarize the predicted TCs by various models, Fig. 2 shows the temporal trend of ∆Cp (Fig. 2a), R_max_ (Fig. 2b), and V_f_ (Fig. 2c) for FL and SWFL. The orange triangles represent the percent change of the median (50th percentile) value of the TC characteristics from the current climate (twentieth century) value, while red and green triangles represent the trends of the 75th percentile and 25th percentile values. For example, when considering 1% coastal flood and its resulting damage on structures, the trend of the 75th percentile and higher values plays a more significant role than those for the 25th and 50th percentiles. On the other hand, the trend of the 25th percentile value has more impact on the more frequent (10–20%) flood.

The trends over FL and SWFL predicted by the CAM5-NASHM, HADGEM-KE, and GFDL-KE models are pretty consistent due to the large number of tracks these models produced. Those predicted by the CESM, HiRAM, CAM5-Happi20, and FSUGSM-WRF, which contain fewer tracks, show more variation between the FL and SW FL scales. This suggests decreased uncertainty in TC prediction on regional and local scales as the number of TCs in the dataset increases. CAM5-NASHM and HADGEM-KE results show relatively minor changes over the twenty-first century, while CESM results do not contain R_max_ and show excessive changes in ∆Cp. GFDL6-KE and FSUGSM-WRF results show the most significant changes over the twenty-first century in SWFL. The median values of (∆Cp, R_max_, V_f_) of the FSUGSM-WRF results changed by (45%, −25%, 22%) respectively, while the GFDL6-KE results changed by (20%, 5%, −15%) respectively. Interestingly, the 75th percentile values changes were (20%, 15%, −15%) for the GFDL6-KE results while (65%, 20%, 35%) for the FSUGSM-WRF results.

### Design of scenarios for coastal flooding analysis

Based on the results presented in "Selection of tropical cyclones for the twenty-first century", we used the TCs predicted by one global climate model (CAM5.1) without downscaling and three global models with downscaling (HiRAM-GFDL, GFDL6-KE, and FSUGSM-WRF) to investigate the compound coastal inundation due to TCs and SLR in SWFL during the twenty-first century. HiRAM-GFDL has a 50-km resolution. KE (Kerry Emanuel’s deterministic-statistical model)^41^ was applied to numerous global climate models^35,36^ and generated hundreds to thousands of TCs for the FL and SWFL, but here we consider the more recent GFDL6-KE results for simplicity. On the other hand, FSUGSM-WRF generated TCs with a 12-km WRF regional climate model^37,40^ for the Southeastern US and contained TCs for more scenarios than any other model. FSUGSM-WRF predicted 60 TCs for FL during 1982–2009 with seven landfalls in the SWFL domain (Fig. 1c), which compared well with the 64 historical TCs and seven landfalls during the same period for FL and SWFL, respectively. It is noted that FSUGSM’s cyclone detection algorithm only recognized hurricanes with Cat-1 intensity or higher. The current climate 1% flood maps for SWFL based on FSUGSM-WRF predictions and historical TCs compared well^53^_._

Sixteen simulation scenarios were designed (Table 1) for coastal flood analysis. For FSUGSM-RCP4.5 scenarios, negative and positive AMO phases were considered, while only the positive AMO phase was considered for the RCP8.5. TCs for the current climate (CC) and late twenty-first century were predicted by all the models, but only FSUGSM-WRF predicted TCs for the early twenty-first century. SLR values include 0 and 0.3 m (99th percentile) for the early period and 1 m (83rd percentile) and 2 m (99.7th percentile) for the late period, based on predicted Global Mean Sea Level (GMSL)^2^. For simplicity, we used the GMSL instead of the RSL (Relative Sea-Level) at Naples and Fort Myers (~ 1.15 m for 50th percentile of 1 m GMSL scenario and 2.55 m for 50th percentile of 2 m GMSL scenario) for this sensitivity study. Zero SLR values are included for the early and late twenty-first century to allow assessment of the effect of SLR vs. that of TCs. Based on the TCs for each scenario, JPM-OS allows the generation of an optimal TC ensemble of 190 TCs for flood simulations by the coastal models.

### 1% coastal flood hazard in twenty-first century

**Figure 3:**
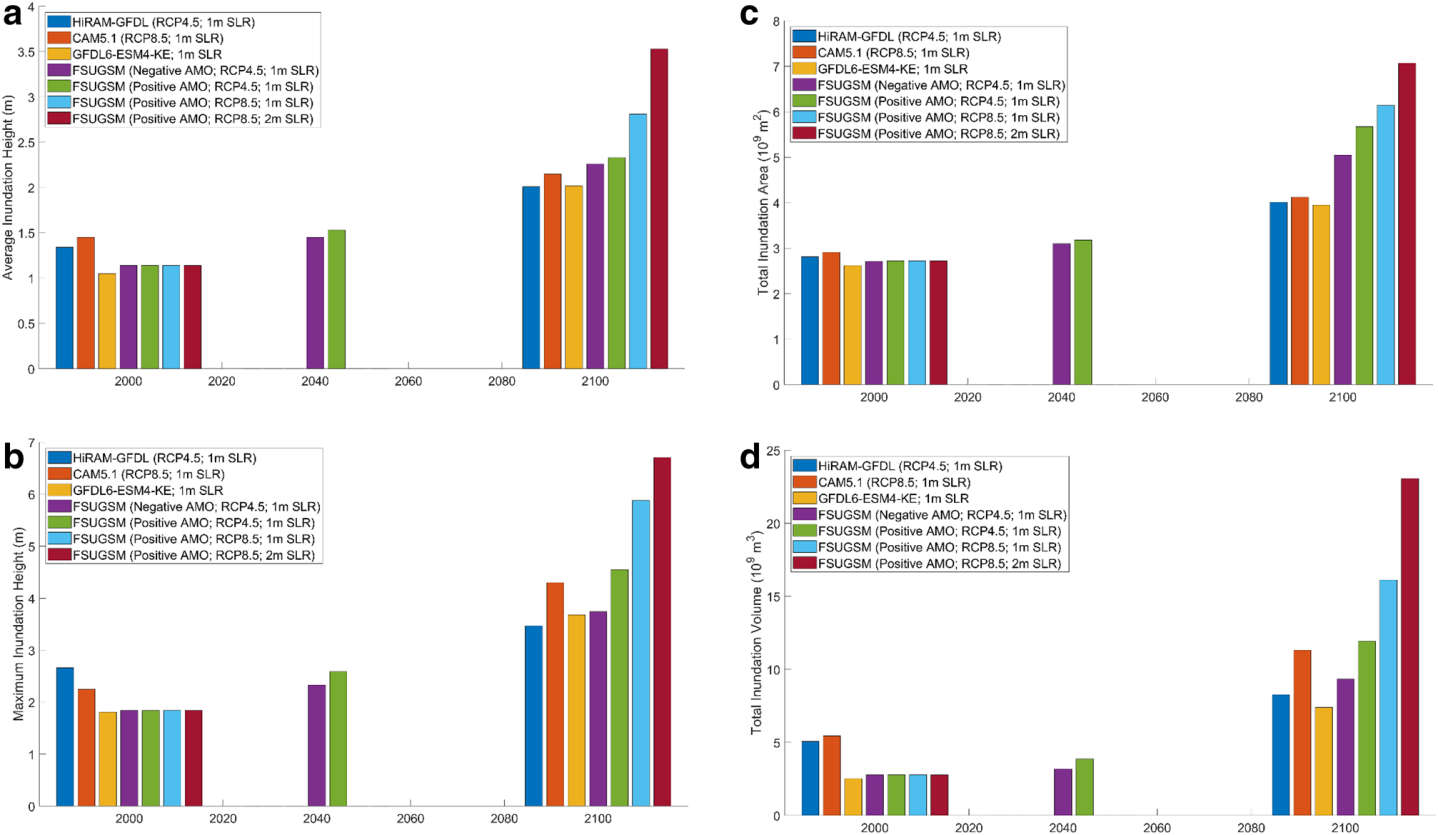
(**a**) Average Inundation Height (*AIH*) of the 1% annual chance flood hazard in SWFL. (**b**) Maximum Inundation Height (*MIH*) of the 1% annual chance flood hazard in SWFL. (**c**) Total Inundation Area (*TIA*) of the 1% annual chance flood hazard in SWFL. (**d**) Total Inundation Volume (*TIV*) of the 1% annual chance flood hazard in SWFL.

As shown in Fig. 3, the inundation metrics (*AIH, MIH, TIA,* and *TIV*) of the 1% annual chance coastal flood in the early twenty-first century increase slightly over those for the current climate (Fig. 3). The inundation metrics increase significantly by 2100 due to increasing TC intensity and SLR. The current climate inundation metrics from the GFDL6-ESM4-KE and FSUGSM-WRF agree well, while those from HiRAM-GFDL and CAM5.1 show slightly higher values of *MIH* and *TIV*. For the late twenty-first century, all models predicted similarly high inundation metrics for RCP4.5 with 1 m SLR. The inundation metrics increase further from negative to positive AMO phase, from RCP4.5 to RCP8.5, and SLR from 1 to 2 m.

Table 2 shows the percent change of inundation metrics relative to the perspective average current climate values. The inundation metrics increase significantly from the current climate to 2100, following the ascending order of HiRAM-GFDL, CAM5.1, GFDL6-ESM4-KE, and FSUGSM-WRF. The inundation metrics in 2100 predicted by the four different models are comparable and consistent. Within the FSUGSM-WRF results, inundation metrics increase for positive AMO, RCP8.5, and SLR. The GFDL6-ESM4-KE-L21and FSUGSM-WRF-L21-N produced similar results showing a 2.4-fold increase in maximum inundation height and a 44–84% increase in total inundation area. For the worst case, FSUGSM-WRF-L21-8.5 with 2 m SLR, total inundation area will increase by 1.58 times while maximum inundation height could increase by three times the current climate value. These results demonstrate the sensitivity of future coastal inundation to TC, SLR, RCP, AMO, and climate and downscaling models. It is noted that, according to the latest IPCC Assessment Report (AR6)^57^ the 2 m SLR in 2100 is highly unlikely.

**Table 2:** Percent increase of inundation metrics from current climate to 2100 for four different models. These values are calculated as follows: $Rate\, of\, increase\, of\, X \left(\%\right)=({X}_{2100}-avg(X)/avg({X}_{2000})$.

	HiRAM-GFDL RCP4.5 1 m SLR	CAM5.1 RCP8.5 1 m SLR	GFDL6-ESM4-KE RCP4.5 1 m SLR	FSUGSM- WRF AMO- RCP4.5 1 m SLR	FSUGSM- WRF AMO+ RCP4.5 1 m SLR	FSUGSM- WRF AMO+ RCP8.5 1 m SLR	FSUGSM- WRF AMO+ RCP8.5 2 m SLR
AIH (%)	68	79	68	88	94	134	194
MIH (%)	75	120	78	80	125	200	238
TIA (%)	46	50	44	84	107	124	158
TIV (%)	140	228	114	171	246	367	568

### Evolution of 1% inundation return period

Comparing the 1% annual chance *TIV* for the FSUGSM-WRF RCP8.5 in 2100 with the *TIV* for the current climate, it is found that the 100-year *TIV* for the current climate could have a 3-year return period by 2100. The current climate's 0.2% annual chance TIV could have a return period of 5 years by 2100. However, the 2 m SLR in 2100 is highly unlikely according to the latest IPCC AR6^57^.

### Evolution of local 10–1000 year flood elevation

Based on the CAM5.1, HiRAM-GFDL, and FSUGSM-WRF results, we present the flood elevation for CC (Current Climate), 2020–2040, and 2080–2100 at a few selected land site (Estero, Naples City Hall, and Marco Island) as well as the storm surge at the Naples tide gauge station which is in the open water (see Fig. 1c for site locations). For CC, the 100-year flood agrees well with that obtained by analyzing Naples's historical water level data. However, the flood elevations at land sites vary significantly due to local storm surge and land conditions. Therefore, it is not feasible to use the Naples surge with a bathtub model (applying the Naples surge over the entire flood plain) to estimate the flooding over the large flood plain.

**Figure 4:**
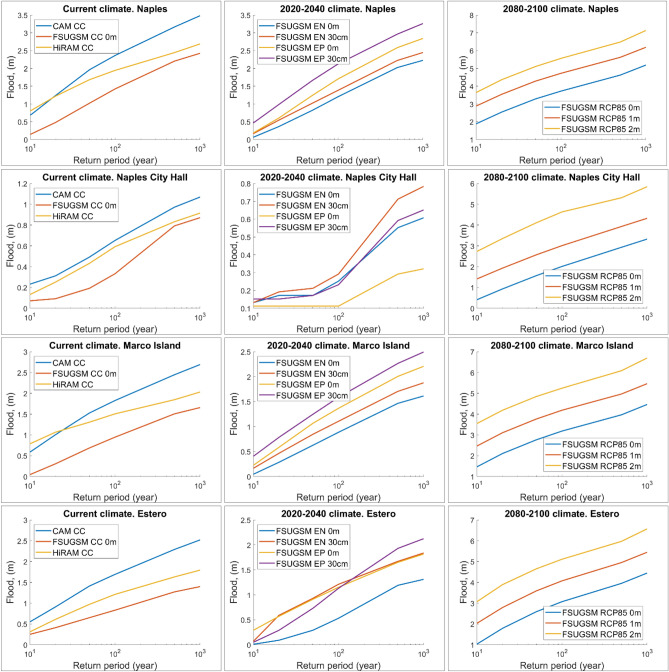
The water level at Naples tide gauge (top row), and inundation height at Naples City Hall (second row), March Island (third row), and Estero (bottom row) during current climate (left column), Early twenty-first Century (middle column), and late twenty-first century (right column). Predictions by CAM5.1, HiRAM-GFDL, and FSUGSM-WRF for the current climate are presented. Future inundation heights are based on FSUGSM-WRF results.

Figure 4 shows that the inundation height increases significantly with time and return period and climate models and scenarios. While the 100-year inundation is the foundation for FEMA to collect flood insurance, it may be prudent for communities to use 500-year inundation for coastal resilience planning. Coastal inundation heights at the selected sites show slight variation between the CC and the 2020–2040 period but significantly increase by 2080–2100. The alarmingly high inundation heights, albeit with uncertainty, warrant immediate attention and adaptation planning by various neighborhoods in the coastal communities.

### Sensitivity of future 1% coastal inundation to SLR

The FSUGSM-RCP8.5 scenarios with 0, 1, 2 m of SLR were used to examine the effect of SLR on the 1% inundation maps. SLR was found to play a dominant role at the inland boundary. Near the coast, storm surge accounts for 70–80% of the total inundation (1 m SLR) or 30–70% (2 m SLR). Therefore, TCs cannot be ignored in developing future coastal flood maps. The effect of SLR on inundation was found to vary significantly over the floodplain due to the non-linear interaction between the storm surge, wave, tide, and SLR. A change in water depth due to SLR can induce non-linear changes in the storm surge, tide, and waves and non-linear changes in the flood propagation as friction is reduced. For example, near the shoreline of Rookery Bay (south of Naples) and Charlotte Harbor (north of Estero), inundation is increased by 1.6 m due to a 1 m SLR. This shows that SLR-induced coastal inundation can be amplified or reduced by the surge-SLR interaction depending on location. Hence coastal flood map produced using a “bathtub” model^58,59^, which adds the SLR value uniformly throughout the floodplain or onto a CC flood map, is highly inaccurate.

### Effect of precipitation

**Figure 5:**
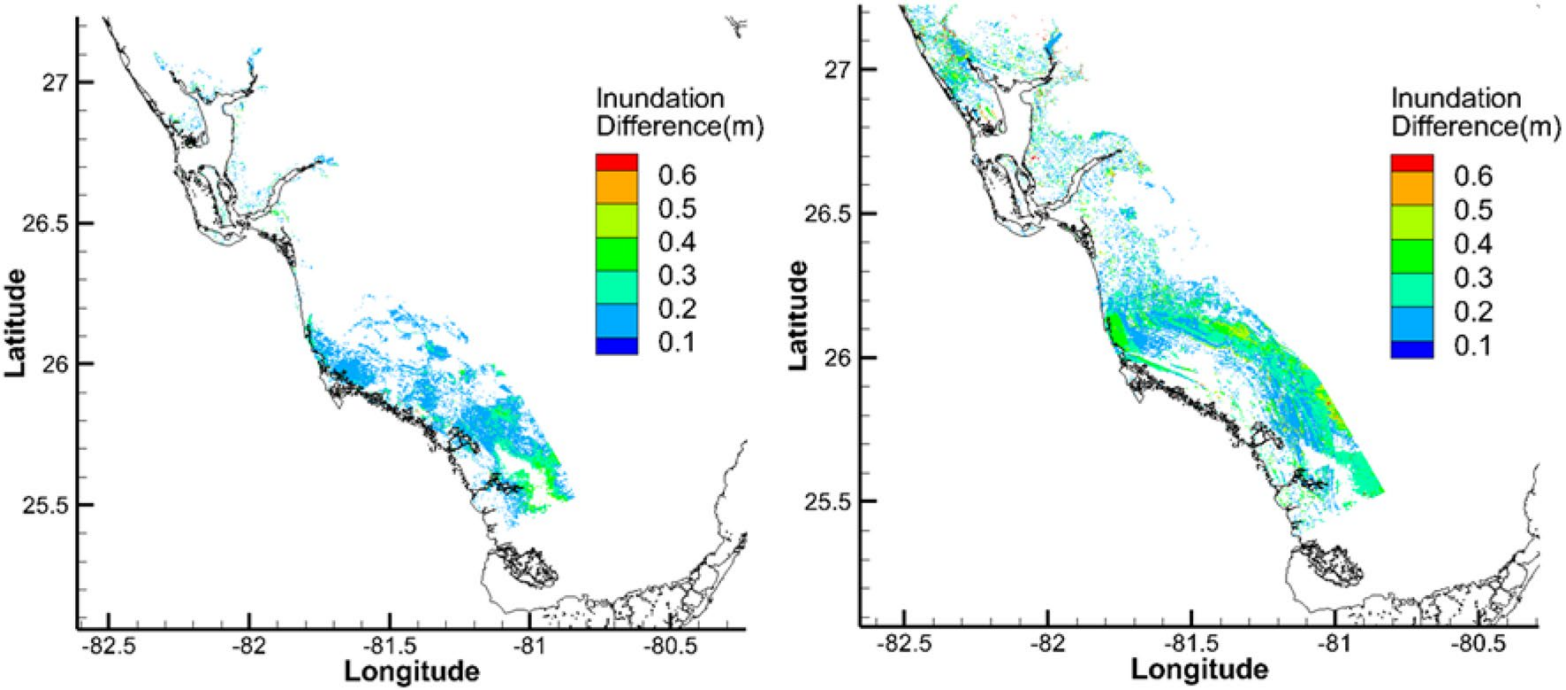
Differences between the 1% inundation with and without precipitation for (left) current climate, (right) RCP8.5-FSUGSM with 2 m SLR. Figure was generated using Tecplot 360 2018 R1 (www.tecplot.com).

For CC, with the 1% precipitation at Naples of 16 inches over three days, *TIV* and *TIA* increased by 13% and 12%, while *AIH* and *MIH* increased by 1 cm and 5 cm, respectively. For 2080–2100, the 0.2% rainfall data were utilized to reflect the increase in precipitation in future climates. With the 0.2% precipitation of 22 inches in 3 days at Naples, the 1% inundation map for RCP8.5-FSUGSM with 2 m SLR showed that the *TIV* and *TIA* increased by 3%, while *AIH* and *MIH* increased by 2 cm and 5 cm, respectively. Figure 5 shows the differences of 1% inundation maps with and without precipitation for the two scenarios. While changes in *AIH* and *MIH* due to precipitation are within a few centimeters, precipitation could increase the local flooding by about 30 cm under CC and future.

### Effect of changing land use features

The effect of (climate-induced) changing land use features on future flooding was addressed by assuming the mangroves will replace all marshes in the floodplain by 2100. The resulting future flood maps were found to show negligible differences, likely due to the use of the 2D surge-wave model, which represents the vegetation with an empirical bottom friction coefficient. Therefore, future study should use a three-dimensional vegetation-resolving surge-wave model, e.g., CH3D-SWAN^13–15^, coupled to a watershed model, with extensive vegetation data (type, distribution, height, stem density, leaf area index, stem size, rigidity, inland migration, etc.) to produce more accurate flood maps and to quantify the value of coastal wetlands in reducing coastal flooding damage in the region^8,60^ to enhance coastal resiliency and wetland restoration.

### Comparison of Coastal Inundation Metrics obtained by different statistical methods

As shown in Yang et al.^53^, the 1% coastal inundation metrics (AIH, MIH, TIA, TIV), in units of ([m], [m], [10^9^m^2^], [10^9^m^3^]), respectively, for SW FL in current climate are (1.06, 2.41, 2.65, 2.82) according to the JPM-OS. Those produced using the MCLC, driven by the TC associated with the 1% Naples water level at 50 percentile confidence level, are (0.27, 2.14, 3.11, 0.73), while those driven by the TC associated with the 1% TIV for the model domain are (0.27, 2.13, 2.83, 0.91). Therefore, the MCLC resulted in much lower AIH and TIV but slightly lower MIH and slightly higher TIA. Inundation metrics obtained by imposing the 1% Naples water level along the open boundary of the coastal model, as in a bathtub model, are less than 50% of those produced by the JPM-OS using TC ensemble for the current climate.

## Discussion

The compound effects of TCs and SLR on coastal flood hazard in a large coastal flood plain in SWFL in the late twentieth (1980–2000), early-twenty-first (2020–2040), and late-twenty-first (2080–2100) century, under RCP4.5 and RCP8.5 greenhouse gas concentration scenarios, were simulated by a coupled surge-wave model for all possible TCs with probabilistic SLR values. The 1% coastal inundation in SWFL is sensitive to the TCs, SLR, RCP scenarios, AMO phases, precipitation, and climate and downscaling models. We further discuss the sensitivity of coastal inundation in SWFL to TCs, SLR, precipitation, and how SLR and TCs are combined in the coastal model simulations.

As shown in Table 2, 1% coastal inundation in SWFL increases significantly from the current climate to 2100. The increase rates for all inundation metrics predicted by four different models show a similar temporal trend. CAM5.1 is a global climate model with a high resolution of 25 km. HiRAM-GFDL is based on the global HiRAM model but downscaled by the regional GFDL hurricane model. GFDL6-ESM4-KE is based on the global model GFDL6-ESM4 with downscaling by the KE model. Finally, FSUGSM-WRF is based on the global atmospheric model FSUGSM forced with SST and surface fluxes of the CanESM2^61^ global atmosphere–ocean climate model and downscaled by the regional climate model (RCM) WRF with a 12-km grid. TCs (of 75th percentile and higher) predicted by GFDL6-ESM4-KE and FSUGSM-WRF show a significant increase in central pressure deficit and Rmax by 2100 and resulted in similar results 1% coastal inundation for the current climate and 2100.

The relative importance of TC characteristics in affecting coastal flooding can be readily analyzed for a single TC. It is known that increased intensity would increase the damage during most historical TC such as Katrina and Sandy. Hurricane Katrina, a Cat-3 hurricane at landfall, caused catastrophic damage due to its huge size, slow speed, and a particular track which caused the collapse of the New Orleans Flood Protection System. Hurricane Charley, a Cat-5 TC, caused minor damage in Florida due to its fast speed and small size. Hurricane Sandy caused a dramatic loss in New York and New Jersey because of its sheer size, slow speed, and a particular track plus coincidence of landfall with high tide.

On the other hand, the 1% flood is the cumulative result of 20,625 total potential TCs which depend on the climate and downscaling models. For example, 1% flood due to the FSUGSM-WRF TCs is found to increase dramatically from CC to 2100 due to a 60–75% increase in the 75th percentile pressure deficit, despite a 20% increase in Rmax and 40% increase in forward speed. On the other hand, the GFDL6-KE TCs resulted in a significant increase in coastal inundation due to a 20% increase in 75th percentile pressure deficit and Rmax but a 10% reduction in forward speed. Moreover, the relative importance of TC characteristics in affecting the increase in future coastal flooding is highly dependent on the location because the 1% flood does not result from a single TC. Therefore, a comprehensive analysis of the relative importance of TC characteristics on probabilistic floods requires a detailed analysis of future coastal floods during all of the optimal TCs.

Although we did not compare the TCs and coastal inundation based on all CMIP5 and CMIP6 climate models and downscaling models, this study suggests that downscaling with KE model (e.g., GFDL-ESM4-KE) and high-resolution RCM (e.g., FSUGSM-WRF) can improve the prediction of TCs by global climate models. NASHM^38^ predicted TCs were successfully used to calculate the 1% flood in New Jersey and New York^8^. Both KE and NASHM can generate hundreds or more TCs for SWFL to enable more robust results. Higher-resolution climate models with a 4–8 km resolution can be available soon to provide a more accurate simulation of future TCs. Further reduction of the uncertainties of probabilistic coastal inundation prediction over a large coastal flood plain can be achieved by a comprehensive study on the sensitivity of TCs and coastal inundation to more global climate models with different complexity (e.g., cloud physics) and resolution, without and with downscaling by a variety of models (e.g., KE, NASHM, GCM) of varying physics and resolution.

Tropical cyclones and sea-level rise can significantly change the sandy beaches and coastal wetlands (marshes and mangroves) along the southwest Florida coast. A single intense tropical cyclone could result in dramatic beach erosion and change of shoreface morphology^62^. In addition, tidal wetlands are highly vulnerable to end-of-century submergence, resulting in extensive losses of habitats^63^. Mangroves in SW FL sustained significant damage due to storm surge and ponding during Hurricane Irma^64^. While a process-based mangrove migration model is being developed, we greatly simplified the analysis by assuming the mangroves will completely replace all marshes in the floodplain by 2100. The resulting future flood maps were found to show negligible differences, likely due to the use of the 2D surge-wave model, which represents the vegetation with an empirical bottom friction coefficient. An ongoing study is using the three-dimensional vegetation-resolving surge-wave model, CH3D-SWAN^13,55,60^, coupled to a watershed model, a stormwater model, and a mangrove migration model, with extensive vegetation data (type, distribution, height, stem density, leaf area index, stem size, rigidity, and inland migration, etc.) to produce more accurate flood maps for the twenty-first century and to quantify the ecosystem service value of coastal wetlands in reducing coastal flooding damage in the region to enhance coastal resiliency and wetland restoration. To further improve the accuracy of flood simulation, the horizontal resolution of the coastal model could be refined to 5–10 m throughout the model domain^60^.

Due to the overwhelming impact of TCs and SLR in the study region, we focused on the compound flooding due to TCs and SLR while simplifying the effect of precipitation and hydrologic flow by using NOAA precipitation data. As a result, our predicted coastal flood hazard for the region should be more robust than that produced by a hydrologic model^65^ forced by the coastal water level (at sparsely located tide stations) representing the combined effect of TCs and SLR^66^. Forcing the hydrologic model with a specified coastal water level is similar to the bathtub approach which gives 50% lower coastal inundation results over the floodplain.

## Summary

The compound effects of TCs and SLR on coastal flood hazard in a large coastal flood plain in southwest Florida in the late twentieth (1980–2000), early-twenty-first (2020–2040), and late-twenty-first (2080–2100) century, under RCP4.5 and RCP8.5 greenhouse gas concentration scenarios, were simulated by a coupled surge-wave model for all possible TCs with probabilistic SLR values. Spatially and temporally varying coastal inundation over the coastal flood plain is sensitive to the TCs, SLR, RCP scenarios, AMO phases, precipitation, and climate and downscaling models.

For the current climate, inundation metrics obtained using TCs from GFDL6-KE and FSUGSM-WRF agree well with each other and those obtained with the historical TCs. Both HiRAM-GFDL and CAM5.1 produced slightly higher coastal inundation for the current climate, perhaps due to the coarse resolution. By 2100, probabilistic coastal inundation hazard in SW Florida as measured by *AIH, MIH, TIA,* and *TIV* predicted by the four models show a similar and consistent temporal trend. In the twenty-first century, (*AIH, MIH, TIA, TIV*) could increase by (68–88, 75–120, 44–84, 114–228) percent respectively from the late twenty-first century values. Positive AMO results in (94, 125, 107, and 246) percent potential increases, RCP8.5 could increase the metrics by (1.34, 2, 1.24, 3.67) times, respectively, and 2 m SLR would lead to increases of (1.94, 2.38, 1.58, 5.68) times. By 2100, (*AIH, MIH, TIA, TIV*) could become (2.94, 3.38, 2.58, 6.68) times of their respective values in the current climate, with the unlikely high 2 m SLR. These findings can inform the coastal community of potential future coastal inundation hazards, which is essential for adaptation and resilience planning.

To reduce the uncertainties in coastal inundation associated with the TC prediction by climate models, further research is needed to compare the TCs predicted by high resolution (less than 25 km) global climate models without downscaling and with downscaling by different downscaling models for current and future climates. This will allow the separation of uncertainties of climate models and downscaling models. Higher-resolution climate models and downscaling models which generate more TCs in the coastal region within a 20-year scenario should be preferred. SLR values from the latest IPCC Assessment Report^57^ should be used. Three-dimensional vegetation-resolving coastal surge-wave models with evolving land use features (mangroves, marshes, and buildings) should be used to improve the accuracy of the coastal inundation simulation. TC-induced flood damage to local infrastructures should be estimated for current and future climate scenarios to develop flood mitigation strategies.

Last but not least, the new paradigm for developing future probabilistic coastal inundation hazard maps, which is more appropriate for the twenty-first century than the existing paradigm^27^, can be applied to other coastal regions throughout the world. This paradigm is currently being enhanced by using a vegetation-resolving three-dimensional surge-wave model coupled to a mangrove model, a stormwater model, and a watershed model. Results of this study are informing coastal adaptation planning by local communities.

## Supplementary Information

Supplementary Information.
