# Supplementary material for: A sensitivity study of rising compound coastal inundation over large flood plains in a changing climate
Source: Sci Rep. 2022 Mar 1;12:3403. doi: 10.1038/s41598-022-07010-z (PMC8888567; doi:10.1038/s41598-022-07010-z)
Supplement: Supplementary file 1 — Supplementary Information. [file 41598_2022_7010_MOESM1_ESM.pdf]

Supplemental Information (SI)

SI-1 TC characteristics based on various climate model predictions and historical data

Figure SI-1a and b show ΔC_p values for landfalling TCs in FL and SW FL predicted by the above-mentioned climate and downscaling models as well as from historical data. ΔC_p values predicted by various climate and downscaling models for FL (Figure 2a) and SW FL (Figure 2b) generally increase from 20th century to late 21st century, except those obtained by CAM5.3 and NASHM. FSUGSM-WRF and GFDL6-KE results show significant increase in ΔC_p over the 21st century. According to the FSUGSM and CAM5.1 results for the late 21st century, ΔC_p and R_{max} for RCP8.5 are lower than those during RCP4.5, perhaps due to stabilization of the upper atmosphere due to excessive warming. ΔC_p and R_{max} also show slight increases during positive AMO phase.

Figure SI-1c and d show R_{max} values for landfalling TCs in FL and SW FL predicted by the climate and downscaling models and from historical data. R_{max} predicted by GFDL5-KE, GFDL6-KE, and FSUGSM-WRF show increase from 20th to 21st century, while the other models show little change over time. R_{max} is unavailable from CESM and HiRAM.

Figure SI-1e and f show V_f values for landfalling TCs in FL and SW FL predicted by the climate and downscaling models and from historical data. V_f predicted by HiRAM and GFDL6-KE show slight decrease in the 21st century, while predicted values by CESM, CAM5.3-NASHM, and FSUGSM-WRF show slight increase. Those predicted by the other models show little change in V_f over time. V_f has been found to be a less important characteristic in affecting coastal inundation than ΔC_p and R_{max}.

While FSUGSM-WRF results show more changes in ΔC_p and R_{max} during the 21st century than the GFDL-KE results, it should be noted that FSUGSM-WRF-E21-Z, FSUGSM-WRF-L21-Z results cover only 20-year span each. By combining the FSUGSM-WRF-E21-Z and FSUGSM-WRF-L21-Z datasets together to create FSUGSM-E21 and FSUGSM-L21, the changes in ΔC_p and R_{max} are slightly reduced and less different the GFDL-KE results. Interestingly, in comparison to the historical ΔC_p and R_{max}, both GFDL-KE and FSUGSM-WRF predicted lower ΔC_p but larger R_{max} for the 20th century. Changes in TC directionality did not show significant changes in the 21st century, hence are not plotted here.

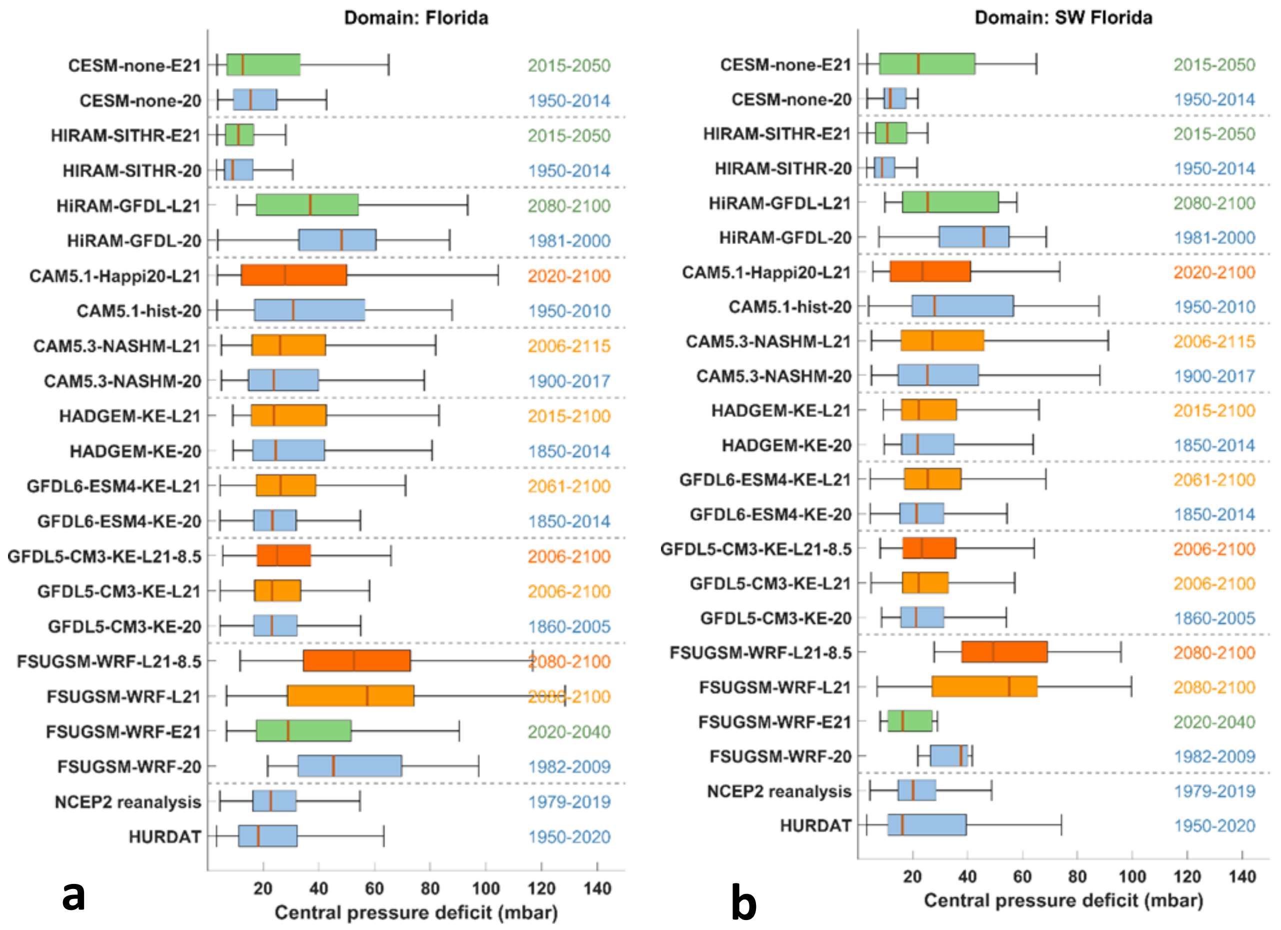

Fig SI-1 (a,b). ΔCp values of landfalling TCs in FL and SW FL. Models predicted TC datasets are named as W-X-Y-Z where W is the climate model, X is the downscaling model, Y is the data period (20=20th century, E21=early 21st century, L21=late 21st century), and Z is the AMO (P=positive, N=negative) or RCP scenario (4.5 unless it is stated at 8.5). Time spans of these datasets are shown in the right column. For each box, the central mark indicates the median, bottom/top edges are the 25th and 75th percentiles, respectively. The whiskers extend to the most extreme data points not considered outliers, and the outliers are shown individually with a red circle symbol.

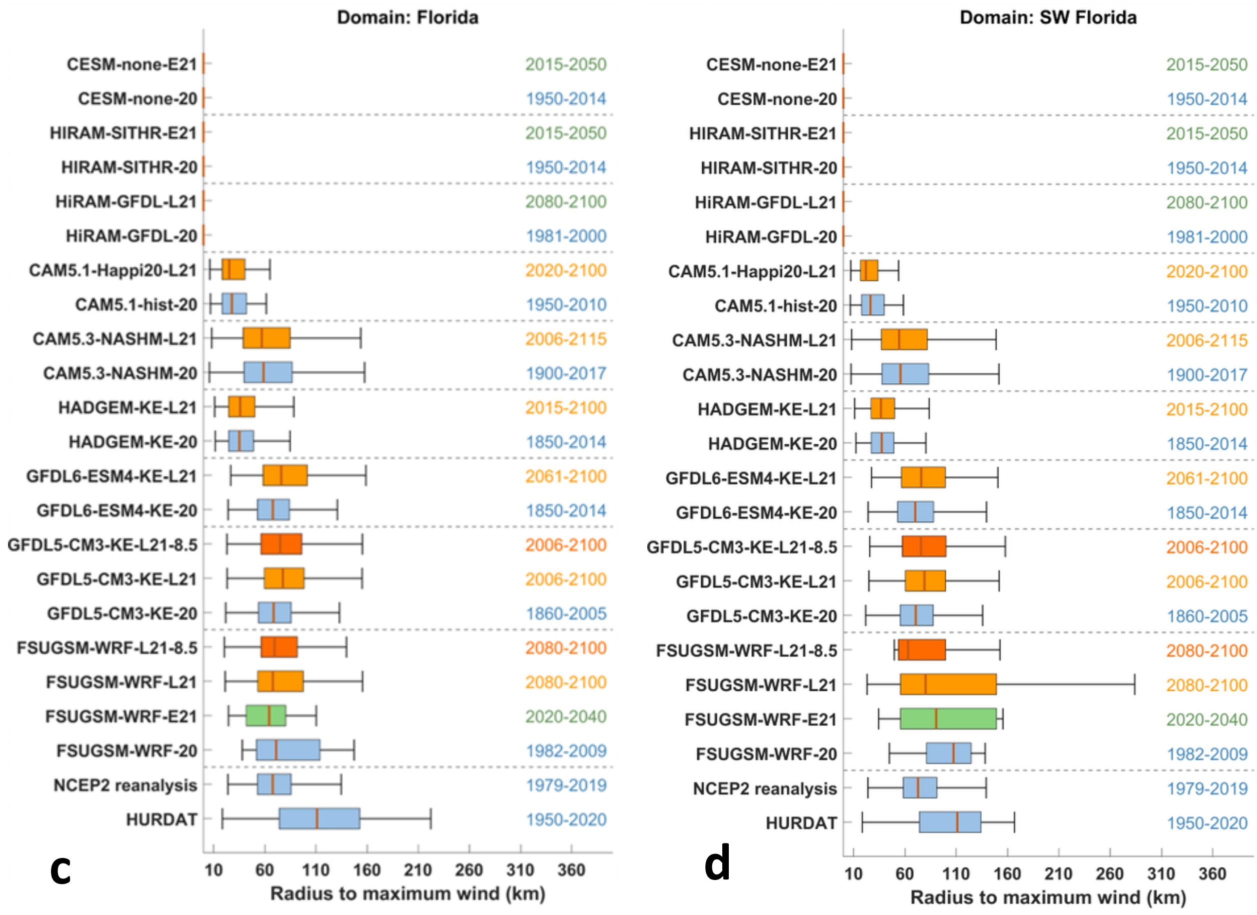

Fig SI-1 (c,d). R_{max} values of landfalling TCs in FL and SW FL (R_{max} data does not exist for CESM and HiRAM datasets).

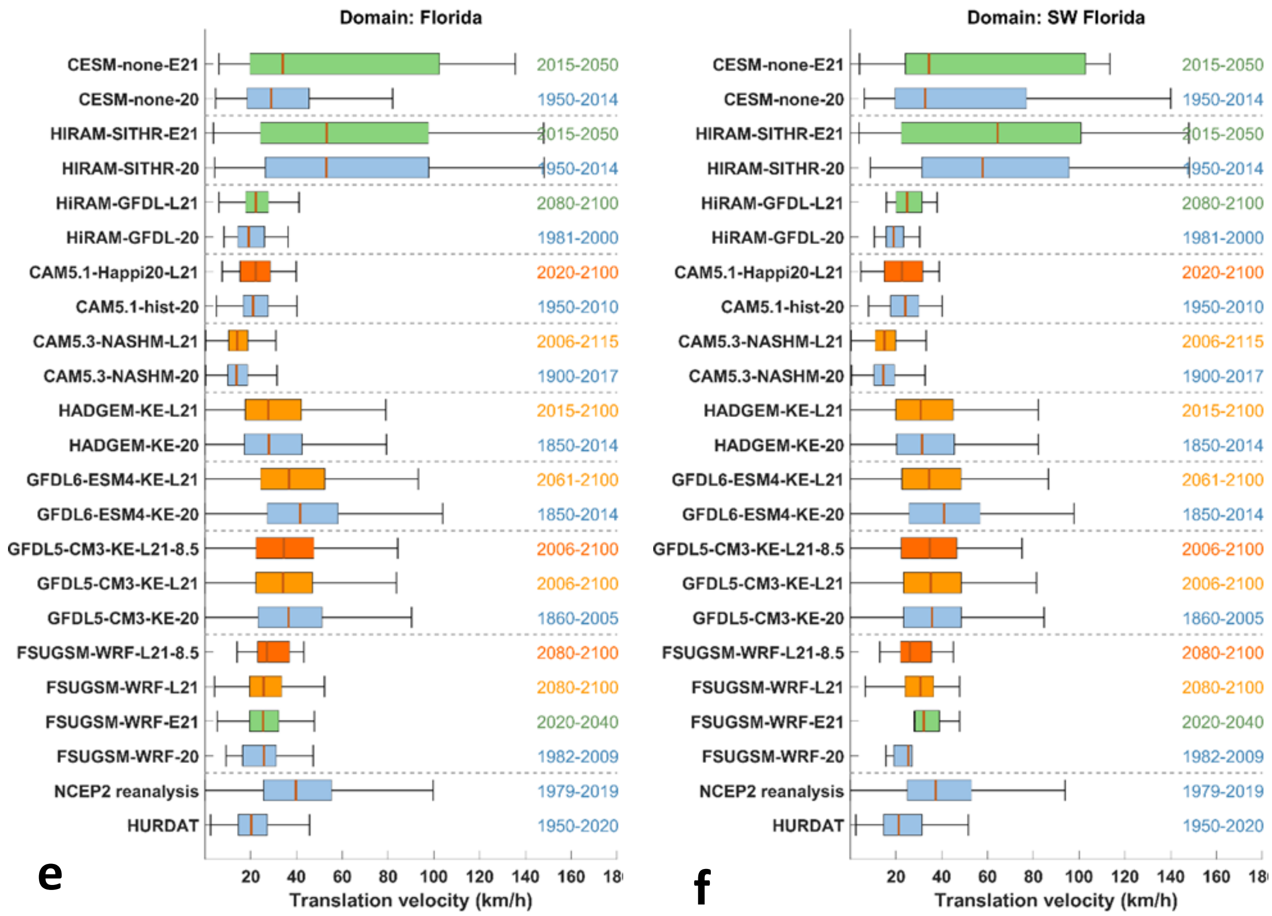

Fig SI-1 (e,f). V_t values of landfalling TCs in FL and SW FL.
